# In vivo CAR T cell therapy against angioimmunoblastic T cell lymphoma

**DOI:** 10.1186/s13046-024-03179-5

**Published:** 2024-09-14

**Authors:** Adrien Krug, Aymen Saidane, Chiara Martinello, Floriane Fusil, Alexander Michels, Christian J. Buchholz, Jean-Ehrland Ricci, Els Verhoeyen

**Affiliations:** 1 Université Côte d’Azur, INSERM, C3M, 06204 Nice, France; 2 Equipe Labellisée Ligue Contre Le Cancer, 06204 Nice, France; 3 CIRI – International Center for Infectiology Research, Inserm, U1111, Université Claude Bernard Lyon 1, CNRS, UMR5308, Ecole Normale Supérieure de Lyon, Université Lyon, F-69007 Lyon, France; 4 Molecular Biotechnology and Gene Therapy, Paul-Ehrlich-Institut, 63225 Langen, Germany; 5 Frankfurt-Cancer-Institute (FCI), Goethe-University, Frankfurt, Germany

**Keywords:** CD8-targeted virus envelope, CAR T, AITL, In vivo gene therapy, Pseudotyping, Lentiviral vector, T cell lymphoma, Cancer therapy, Preclinical model

## Abstract

**Background:**

For angioimmunoblastic T cell lymphoma (AITL), a rare cancer, no specific treatments are available and survival outcome is poor. We previously developed a murine model for AITL that mimics closely human disease and allows to evaluate new treatments. As in human AITL, the murine CD4^+^ follicular helper T (Tfh) cells are drivers of the malignancy. Therefore, chimeric antigen receptor (CAR) T cell therapy might represent a new therapeutic option.

**Methods:**

To prevent fratricide among CAR T cells when delivering an CD4-specific CAR, we used a lentiviral vector (LV) encoding an anti-CD4 CAR, allowing exclusive entry into CD8 T cells.

**Results:**

These anti-CD4CAR CD8-targeted LVs achieved in murine AITL biopsies high CAR-expression levels in CD8 T cells. Malignant CD4 Tfh cells were eliminated from the mAITL lymphoma, while the CAR + CD8 T cells expanded upon encounter with the CD4 receptor and were shaped into functional cytotoxic cells. Finally, in vivo injection of the CAR + CD8-LVs into our preclinical AITL mouse model carrying lymphomas, significantly prolonged mice survival. Moreover, the in vivo generated functional CAR + CD8 T cells efficiently reduced neoplastic T cell numbers in the mAITL tumors.

**Conclusion:**

This is the first description of in vivo generated CAR T cells for therapy of a T cell lymphoma. The strategy described offers a new therapeutic concept for patients suffering from CD4-driven T cell lymphomas.

**Supplementary Information:**

The online version contains supplementary material available at 10.1186/s13046-024-03179-5.

## Background

Peripheral T cell lymphoma (PTCL) is a challenging complex malignancy and represents 12–15% of all lymphoid malignancies in Western countries and includes over 20 entities. However, chemotherapy regimens that cure many patients with B cell lymphomas have produced very disappointing results in PTCL. One of the most prominent PTCLs is angioimmunoblastic T cell lymphoma (AITL), which is a devastating disease, affecting mostly elderly patients [1, 2]. AITL disease outcome is poor, with an overall 5-year survival rate of 30% upon cytotoxic chemotherapeutic treatment [3, 4]. Optimal management of AITL and PTCL represents an unmet medical need. AITL patients are mostly detected in the later stages of disease, marked by splenomegaly, hepatomegaly and a generalized lympho-adenopathy [5–7]. Other clinical manifestations of AITL are skin rash [8], increase in immunoglobulins, manifestation of autoimmune disease and accumulation of abdominal ascites [5, 9]. The low prevalence of this T cell lymphoma makes it difficult to design and evaluate novel therapeutic strategies.

To address this medical challenge, several preclinical models for AITL have been generated relying on genetic mouse models [10–14] by introducing the recurrent mutations present in the malignant T cells. We previously generated a unique in vivo model for AITL by overexpressing glyceraldehyde-3-phosphate dehydrogenase (GAPDH) exclusively in T cells (mAITL mouse). This is one of the glycolytic enzymes emerging now as a key player in T cell survival, development and function. These mice show clinical and pathological features equivalent to AITL patients [15]. At later age they develop a T cell malignancy, characterized by an abundant tumor environment including for the majority germinal center (GC) B cells and neoplastic T cells that have a T follicular helper (Tfh) phenotype (CD4^+^, PD1^high^, CXCR5^+^, ICOS^+^) equivalent to the Tfh gene signature in human AITL [16]. Moreover, through gene-set-expression analysis it was further confirmed that this murine peripheral T cell lymphoma (PTCL) was equivalent to human AITL [15, 17]. We generated thus a unique new murine Tfh lymphoma model that can compensate for the low prevalence of the striking resembling human AITL and gives the opportunity to evaluate novel therapeutic approaches such as pathway interference or immunotherapies [15, 17].

A recent successful anti-cancer strategy is based on engineered T cells called chimeric antigen receptor (CAR) T-cell therapy [18, 19], which involves changing a patient’s own immune cells to augment the immune response to cancer cells. CAR T cells incorporate a T cell receptor-like structure presenting at its surface a scFv of an antibody that binds to a cancer cell specific antigen [20]. CAR T cells are considered an individualized cell therapy product because it demands harvesting of the patient’s T cells, which are then modified and expanded ex vivo to be re-infused as a living drug. This is a very time-consuming and labor-intensive process resulting in a very costly medical product, which is therefore not available for all patients in need of CAR T cell therapy. Moreover, ex vivo expansion can alter the phenotype and the function of the CAR T cells [21]. This supports the development of in vivo CAR T cell therapy, which would reduce costs and make CAR T cell treatment more affordable for patients in the future.

In vivo genetic modification of T cells though needs a specific gene transfer tool that will only target T cells. Receptor targeting is based on the requirement for a gene-delivering vector to use a specific receptor to enter a cell which leads to cell entry selectivity and thus restricts expression of the gene, in this case the CAR expression, to a specific T cell subtype. The main problem of in vivo gene delivery is the possibility of generating off-target cell lentiviral vector (LV) transduction causing severe health risks and adverse effects [22]. For ex vivo generation of CAR T cells LVs are pseudotyped with envelope glycoproteins (gps), which are not suited for in vivo gene therapy since they are not T cell specific and are quickly inactivated in vivo [23, 24]. Suitable glycoproteins for cell specific targeting can be derived from paramyxoviruses, particularly measles virus (MV) and Nipah virus (NiV) [25]. In both viruses, receptor attachment and membrane fusion functions are separated in two different glycoproteins. Therefore, insertion of a cell targeting protein into the receptor binding glycoprotein (gps) allows retargeted binding to a receptor of choice without affecting fusion function [26]. By simultaneously abolishing natural receptor usage in these gps, gene delivery to non-target (e.g. malignant) cells is abolished [24, 27, 28]. By engineering paramyxovirus glycoproteins, LVs have been generated that deliver genes selectively into distinct cell types, such as T cells, cancer cells, subtypes of neurons or endothelial cells [24, 29–31]. Making use of the distinct surface markers of T-cell subtypes, CD8 for cytotoxic T lymphocytes and CD4 for helper and regulatory T cells, enabled the generation of LVs targeted to specific T-cell subtypes [27, 30, 32, 33]. Some of the molecules used for this cell retargeting purpose are scFvs. The use of ankyrin repeat proteins (DARPins) constitutes an alternative due to their versatility and higher affinity [25]. The ankyrin domains have been selected from library screens to identify DARPins with high affinity for a specific receptor [34]. ScFvs and DARPins have been introduced successfully in MV [35] and in Nipah virus envelope glycoproteins [34] to target oncolytic domains and hematopoietic cells in vitro and in vivo [32, 33, 36]. Using CD8-targeted LVs, we provided proof of concept for the in vivo reprogramming of CAR T cells. A single systemic injection of this vector encoding for an anti-CD19 CAR into immune deficient mice engrafted with a human blood system generated in vivo CD8^+^ CAR T cells, which effectively wiped out human B cells [36]. The in vivo–generated CAR T cells eliminated CD19^+^ B lymphocytes as well as tumor cells [36, 37]. Conversely, CD4-LV mediated the exclusive generation of CD4^+^CAR T cells, which were equally active in eliminating tumor cells [38].

The AITL neoplastic cells are Tfh-like cells derived from CD4 + T cells and express several surface markers targetable by CARs: inducible T-cell co-stimulator (ICOS), programed death 1 (PD-1), cluster of differentiation (CD) CD4, CD30, CD38, CD52 and T cell receptor β-chain constant region 1 (TRBC1) [39]. One of the major challenges is that healthy T cells share mutual antigens with malignant cells, which causes fratricide, a phenomenon where CAR T cells do not only target the malignant T cells but also their’brother’ CAR T cells and healthy T cells [40]. This prohibits adequate CAR T cells expansion to assure therapeutic benefit. To prevent CAR T cell fratricide, we used CD8-targeted LVs for CAR delivery. And considered CAR T cells directed against murine CD4 as a promising strategy for AITL patients since ex vivo generated anti-CD4CAR NK or T cells were able to eliminate human T-cell leukemia and lymphoma in preclinical mouse models [41, 42]. The anti-CD4CAR LVs in the current study recognized specifically CD8 as target receptor and allowed the generation of CD4-directed CAR CD8 T cells in vivo, which eliminated CD4 + malignant T cells in a primary murine AITL lymphoma.

## Methods

### Plasmids

SINHIV-PGK-GFP and SINHIV-EF1a-GFP lentiviral vector constructs are available upon request from E. Verhoeyen.

PsPAX2 encoding for HIVgagpol is available from Addgene (#12,260), VSV-G encoding plasmid was described earlier [43]. The backbone for the CAR construct containing the 4–1 BB costimulatory domain and the CD3zeta signaling domain separated by a peptide T2A for co-expression of the human CD34 epitope was a kind gift from Floriane Fusil (CIRI, Lyon, France).

The anti-mCD4 scFV sequence was a kind gift from Wolfgang Uckert (Berlin, Germany) and was synthesized by Thermofisher Scientific for insertion into EF1α promoter-driven CAR construct using the EcoRI and BsiWI restriction sites for cloning. In this CAR construct, we subsequently changed the EF1α promoter for the PGK promoter by PCR amplification of the PGK sequence using the restriction sites EcoRI and ClaI. The pHnse- Δ18mut-L3-MSE10 was a gift by C.J. Buchholz (Paul Ehrlich institute, Langen, Germany) and was generated by inserting via the SfiI/NotI restriction sites the MSE10-DARPIN, targeting CD8, into the measles virus envelope pHnse-Δ18mut-L3 previously described [31].

### Cell lines and mouse T cells

HEK293T kidney cell line (ATCC CRL-3216) was grown in DMEM medium (Life Technology, Paris, France) with 10% FCS and 50 μg/ml of penicillin/streptomycin (Invitrogen, Fisher scientific).

C57/BL6 T lymphocytes were isolated by negative selection with a cocktail of FITC coupled antibodies as listed: anti-CD11b (Biolegend, 101,206), anti-NK1.1 (BD Biosciences, 553,164), anti-CD122 (BD Biosciences 554,452), anti-Ly6G (BD Biosciences, 553,127), anti-TER119 (Miltenyi, 130–112-719), anti-CD19 (Miltenyi, 130–119-800). After a wash in PBS/2% FCS, the cells were incubated with anti-FITC microbeads from Miltenyi Biotec (Paris, France) (130–048-701) according to the manufacturer’s instruction. T cell were then sorted by autoMACS from Miltenyi Biotec (Paris, France) and cultured in RPMI medium (Life Technology) supplemented with 10% FCS, 50 μg/ml of penicillin/streptomycin (Invitrogen, Fisher scientific, Illkirch, France) and of 50 μM of β-mercapto-ethanol (Sigma-Aldrich/Merck, Darmstadt, Germany), named RPMI complete. Other supplements for activation of T cell are indicated below.

### Production of lentiviral vectors

CD8 receptor targeted LVs (mCD8-LVs) were produced according to our established protocol [28]. Briefly, 293 T cells were transfected using polyethylenimine (PEI) with pHnse-Δ18mut-L3-MSE10 [35] and pCG-FΔ30 [27], the transfer vector encoding plasmid, the packaging plasmid Pax2 in a 5.8/17.5/63/86 ratio. For VSV-G-LV production hCMV-VSV-G plasmid [43], transfer vector and packaging plasmid were transfected in a ratio of 35/100/65. The medium was replaced by OPTIMEM medium (Life technologies, Paris, France) complemented with 1% Pen/Strep and 1% HEPES (Life technologies, Paris, France). Eighteen hours later the supernatants were harvested and clarified through 0.45 μm filter. The supernatant was then concentrated by low speed centrifugation (4 °C, 3000 g, overnight). 100-fold concentrated vector preparations were stored at -80 °C.

### Plck-GAPDH mouse model

Plck-GAPDH mice were generated in our lab by microinjection of the plasmid encoding for GAPDH-V5 into pronuclei of oocytes from C57BL/6 J, as described in our previous study [15]. Mice were bred and maintained under pathogen-free conditions at the local animal facility (C3M, INSERM U1065, Nice, France). At sacrifice, single cell suspensions were prepared from the lymphoma biopsies (spleen, LNs) for further experimentation and analysis. Experimental protocols were approved by the local ethical (SBEA, Nice, France, autorisation N° B0608820).

### Isolation of primary mouse T lymphoma cells

From aged plck-GAPDH mice, which developed AITL lymphoma, we isolated the enlarged spleens and lymph nodes. These hematopoietic tissues were homogenized to single cell suspensions, which were then used for the in vitro transduction experiments.

### Titration by physical particles (HIV p24gag content)

Vectors were titered for physical particles by measuring p24 antigen using an enzyme-linked immune-absorbant assay (ELISA) following manufacturer’s instructions (RetroTek-ZeptoMetrix, Buffalo, NY).

### Transduction of murine T cells

T cells were either activated by the survival cytokines IL-15 and IL-7 at 20 ng/ml in RPMI complete medium for 3 days or through the TCR by precoating of a culture plate with 3 μg/ml of anti-CD3 antibody (BD Biosciences, #567,115) in which the T cells (5E5 cells/ 24-well) were seeded in RPMI complete medium supplemented with anti-CD28 antibody (BD Biosciences, #567,110), and 20 ng/ml IL-2 for 1 day before addition of the vectors at indicated p24 doses. At day 3 and day 6 post-transduction the T cells are analysed by flow cytometry. Half of the medium is replenished every 2 days.

### Co-culture of CAR T cells and macrophages

Bone marrow (BM) was isolated from the tibia of C57BL/6 mice. BM was then put in culture for 5 days to allow differentiation in bone marrow derived macrophages (BMDM) in RPMI GLUTAMAX medium (Life Technology) supplemented with 10% FCS, 50 μg/ml of penicillin/streptomycin (Invitrogen, Fisher scientific—Illkirch, France), gentamycin (Thermofisher, 15,750–045) and murine M-CSF (macrophage colony stimulating factor; Miltenyi, 130–101-704).

In parallel, T cells were pre-stimulated with IL-7 and IL-15 and transduced with mCD8-LVs at equivalent physical particles content (p24). At day 3 post-transduction pGKGFP/pGKCAR expression was analyzed in the T cells. Subsequently the transduced T cells were co-cultured with BMDM in a ratio 1:1 for 72 h and then analyzed by FACS using staining with anti-CD3 APCcy7 (130–102-306) and F4/80 PE (130–116-499) from Miltenyi Biotec (Paris, France) and staining for death cells with DAPI.

### Flow cytometry and antibodies for murine immune cells

Antibodies used for detailed phenotyping or intracellular staining by flow cytometry of murine T cells are listed here and acquired from Miltenyi Biotec (Paris, France): CD3 APCcy7 (130–102-306), CD4 Vioblue (130–118-568); CD8 PEcy7 (130–119-123), PD-1 PE (130–111-800), or BD Biosciences: INFγ APC (554,413) or E-bioscience/ Fisher scientific, Illkirch, France (Perforin PE (12–9392-82), Granzyme B PEcy7 (25–8898-82). For detection of the CAR expression, we relied on the co-expression of human CD34 detected by anti-hCD34 APC from R and D Systems (#FAB7227-10). Cell activation was evaluated by anti-CD69 APC-Cy7 (Miltenyi, 130–103-984).

For intracellular staining of Granzyme B, Perforin and IFNγ, splenocytes were stimulated for 5 h in PMA (phorbol 12-myristate-13-acetate; Sigma, # P8139)/ionomycin (Sigma, # I0634) in the presence of Golgi-stop (BD Biosciences, #555,029) and upon surface staining (anti-CD4 and anti-CD8) cells were fixed and permeabilized using the Cytofix/Cytoperm kit and protocol (BD Biosciences; #554,714). All stainings were detected using a MACSQuant flow cytometer (Miltenyi Biotec, Paris, France). Analysis of the FACS data was performed using MACSquantify Version 2.11 (Miltenyi) and FlowJo Software.` For FACS analysis of splenocytes or T cells, we applied following gating strategy: gating on lymphocyte population in the SSC-A versus FSC-A was performed, followed by gating single cells in an FSC-A/FSC-H plot. Subsequently, a plot SSC-A versus Propidium iodide gating on PI negative cells was performed. Then we gated in SSC-A versus CD3 blot for T cells on the CD3-APCCy7 + cells followed by gating on CD4 + Vioblue or CD8 + PECy7 T cells. Alternatively, upon gating of the PI negative cells, plots for CD4 + Vioblue or CD8 + PECy7 cells were shown on which gating for CD4 or CD8 was performed. For B-cel detectionl we gated on living cells and then on CD19 + in the CD19 versus CD3 plot. For further gating strategies are depicted in the main figures or supplementary figures.

### In vivo treatment of murine AITL mice

Single cell suspensions from the spleen of aged plck-GAPDH mice (> 18 months) with splenomegaly were intravenously injected (1–2 × 10^7^ splenocytes per mouse) into 6- to 8-week old recipient NOD/SCIDγ-/- mice (NSG; Jackson Laboratory, Charles River France, L’arbresle, France; #005557). Since Tfh CD4 + lymphoma cells are not detectable in the blood, recipient mice were sacrificed week 6, 8 and 12 upon tumor cell injection to determine the timepoint of efficient lymphoma engraftment. At week 12 of engraftment, 20 engrafted NSG mice were injected at day 4 and day 1 before vector injection with IL-15 (200 ng) and IL-7 (200 ng). Subsequently 10 recipient mice were injected intravenously with anti-CD8LV encoding GFP (2E6 TU), and 10 recipient mice were injected with anti-CD8LV encoding the anti-CD4CAR (2E6 TU), both under the control of the PGK promoter. All NSG recipient mice were sacrificed at humane endpoint (> 10% weight loss) or before. Single cell suspensions were prepared from the spleen for immunophenotypic analysis by FACS.

### Quantification and statistical analysis

Statistical analysis was conducted using Microsoft excel 2013 and Prism software v6.0 (GraphPad Software, La Jolla, CA, USA). Results are indicated as means (SD) in the figure legends unless indicated otherwise. T-test was used for two-group comparison followed by Mann Whitney test, non-parametric. One-way ANOVA followed by Tukey’s multiple comparisons test was used for multi-group comparison, if the data obey the normal distribution. In the case that the data do not obey the normal distribution, Mann–Whitney test was used for two-group comparison and Kruskal–Wallis test was used for multi-group comparison. The statistical tests applied are indicated in the figure legends. p-values are indicated in the figure legends. A *p*-value < 0.05 was considered to indicate statistical significance.

## Results

### Murine CD8 targeted LVs allow highly selective CD8 T cell transduction

**Fig. 1:**
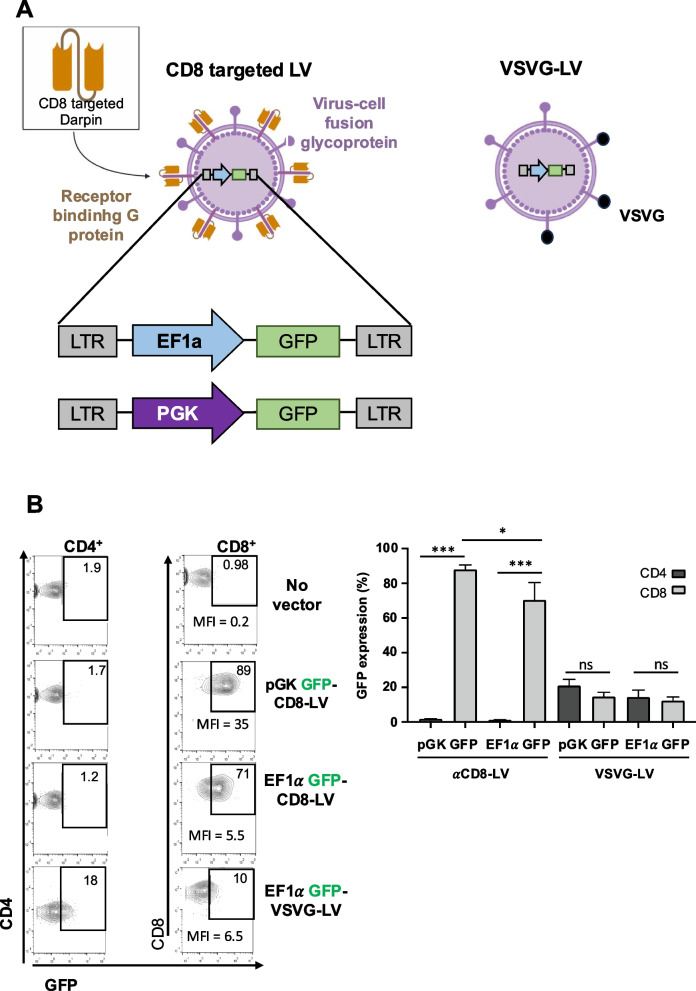
Specific transduction of CD8 + murine T lymphocytes by mCD8-LVs (**A**) Schematic representation of CD8-targeted lentiviral vectors (mCD8-LVs) or VSV-G pseudotyped LVs. CD8-LVs carry at their surface the measles virus fusion protein (F) and the receptor binding H protein fused to a DARPin, specific for mouse CD8. The two lentiviral vector constructs incorporated into these LVs are presented. SIN: self-inactivating; LTR: HIV long terminal repeat; EF1a: elongation factor 1 alpha promoter, PGK: PGK: phosphoglycerate kinase 1 promoter. **B** Transduction of murine splenic T cells after TCR-stimulation was performed with mCD8-LVs (10 ng p24 content) or VSV-G-LVs (2 ng p24 content) carrying the EF1a-GFP or the PGK-GFP expression cassettes Transduction was analyzed by FACS on day 3 post-transduction by gating on CD4 + T cell and CD8 + T cells and summarized in (**C**). Data are represented as mean (SD) (*n* = 3; biological replicates, multiple t-test, *p < 0.05; ***p < 0.001, ns = not significant)

In AITL the malignant cells are CD4 + Tfh like cells. Our objective is to generate CAR T cells that will eliminate these CD4 + T cells from the tumor tissue. For this reason, we are obliged to use LVs that will exclusively transduce the CD8 + tumor infiltrating T cells in order to avoid CAR T cell fratricide and CAR expression by their counterpart malignant CD4 T cells. Previously, we selected a CD8 specific designed ankyrin repeat protein (DARPin) from a DARPin library exposed to the murine CD8 heterodimer receptor. This DARPin (MSE10) was then inserted into the receptor-blinded truncated hemagglutinin (H) from measles virus (MV) [35]. We subsequently produced LVs carrying at their surface the CD8-retargeted MV H and the fusion glycoprotein protein F (mCD8-LV) or conventional LVs pseudotyped with the vesicular stomatitis virus G glycoprotein (VSV-G-LV; Fig. 1A). We employed LVs carrying a GFP reporter cassette driven by an elongation factor 1 α (EF1α) promoter or the stronger phosphoglycerate kinase 1 (PGK) promoter. To prove the selectivity of both vectors for CD8 T cells, we isolated T cells from splenocytes from wild type (WT) C57BL/6 mice since the preclinical mAITL mouse model was generated on a C57BL/6 background [15]. The T cells were activated through the T cell receptor (TCR) with anti-mCD3 and anti-mCD28 antibodies. Subsequently, they were transduced with PGK-GFP or EF1-GFP encoding VSV-G-LVs and mCD8-LVs. We used physical particle titers as determined by p24 content. To emphasized the specificity of the mCD8-LVs we used high levels of viral particels (p24 content = 10 ng), while for the VSV-G-LVs we used a non-saturating condition (2 ng p24). Flow cytometry analysis confirmed a highly selective transduction by mCD8-LVs into CD8 + splenocytes for both promoters used, while the VSV-G-LVs transduced both the CD4 + and CD8 + T cells with equivalent efficiency even a low vector doses (Fig. 1B). Indeed, we achieved 85% and 70% transduction of mCD8 T splenocytes with mCD8-LVs for the PGK and EF1a promoters,respectively, resulting at least in a 50- and 60-fold selectivity for mCD8 versus mCD4 T cells. Importantly, we detected a much higher level of expression (mean fluorescence intensity, MFI) for the PGK promoter than the EF1a promoter construct and no differences in the T cell subpopulations were detected as compared to untransduced T cells (Fig. 1B).

### ***mCD8-LVs allowed high level anti-CD4CAR expression driven by the PGK***_***prom***_*** in mCD8 T cells***

**Fig. 2:**
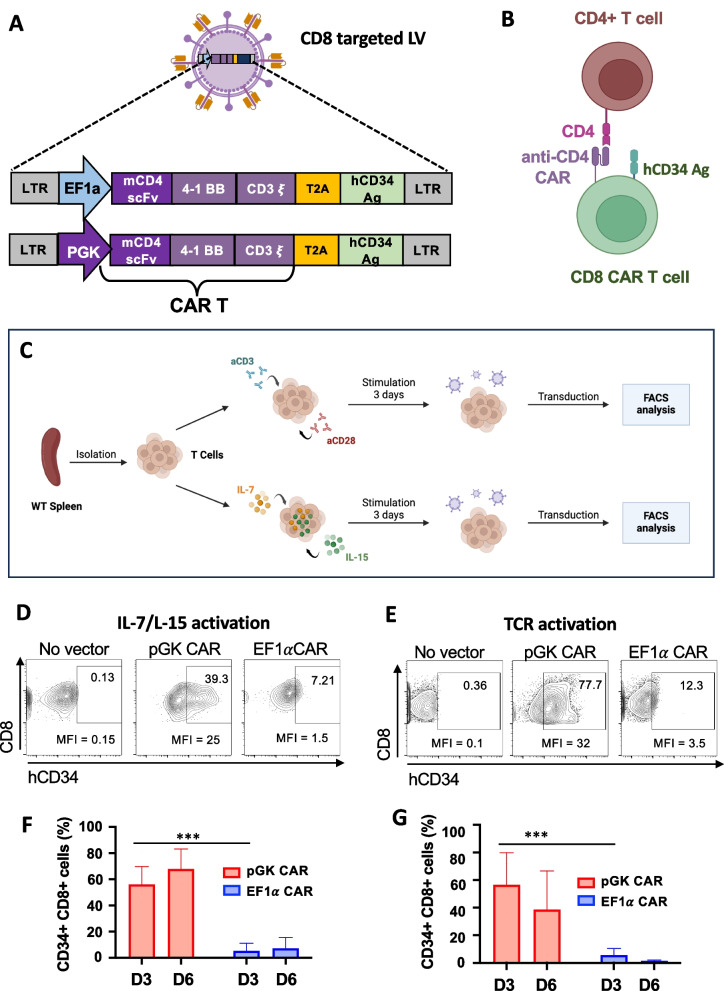
Anti-CD8-LVs show highest murine CD4-CAR expression when driven by the PGK promoter. **A** Schematic representation of the mCD8-LVs and the anti-mCD4 expressing CAR encoding vector constructs. LTR: HIV long terminal repeat; EF1a: Elongation factor 1 alpha promoter, PGK: phosphoglycerate kinase 1 promoter; mCD4scFv: single chain variable fragment of a murine anti-CD4 antibody; 4-1BB: T cell costimulatory domain; CD3ζ: CD3 signaling domain; T2A: peptide permitting co-expression of two proteins; hCD34: epitope recognized by an anti-human CD34 antibody. **B** Schematic representation of the CD8 + cells expressing the anti-CD4CAR binding to CD4 + T cells and co-expressing the hCD34 epitope to facilitate detection by FACS using an anti-hCD34 antibody. **C** Representation of the stimulation and transduction protocols for T cells isolated from WT spleens. Created with Biorender.com. T cells were activated either using anti-CD3 and anti-CD28 antibodies, or IL-7/IL-15 cytokine stimulation for 3 days. Then the T cells were transduced with the indicated vectors at equivalent numbers of viral particles (p24: 10 ng); Created with Biorender.com. At day 3 (**D**, **E**) and day 6 (**F** and **G**) post-transduction, cells were evaluated for hCD34 + expression by FACS analysis. Data are represented (mean (SD) (**F**) PGK-CAR d3 *n* = 6, d6 *n* = 3; EF1a-CAR d3 *n* = 4, d6 *n* = 3 and (**G**) PGK-CAR d3 *n* = 7, d6 *n* = 6; EF1a-CAR d3 *n* = 5, d6 *n* = 3; t-test; ***p < 0.001)

The Tfh-like origin of AITL expresses several surface markers: ICOS, PD-1, CD4, CD30, CD38, CD52 and TRBC1. We chose to direct the CAR toward a CD4 molecule, present on both malignant and healthy CD4 T cells. We next generated two lentiviral constructs encoding for an anti-mCD4 CAR driven by the PGK or EF1α promoters. The CAR consisted of a mCD4 scFv (mAb clone GK 1.5) fused to a mCD8 linker, a transmembrane sequence linked to the cytoplasmic 4-1BB co-stimulatory domain and the CD3ζ T cell receptor (TCR) signaling domain (Fig. 2A). The CAR sequence was linked to a human CD34 epitope sequence by a T2A peptide. The hCD34 epitope facilitates detection of the T cells expressing the CAR via flow cytometry analysis since the resulting transduced mCD8 cells will co-express the anti-CD4 CAR and the hCD34 epitope at their surface (Fig. 2B). T cells were isolated from WT C5BLl/6 splenocytes and activated through the TCR by anti-CD28 and anti-CD3 antibodies or by T cell survival cytokines, IL-7 and IL-15, before transduction as outlined in Fig. 2C. For both stimulations the PGK CAR mCD8-LVs outperformed by far the EF1aCAR mCD8-LVs in transduction levels as well as expression levels indicated as mean fluorescence intensity (MFI) of the CAR expression (Fig. 2D-G).

### ***PGK***_***prom***_*** driven anti-CD4CAR*** + ***mCD8 T cells were expanded and functional in eliminating CD4 T cells***

**Fig. 3:**
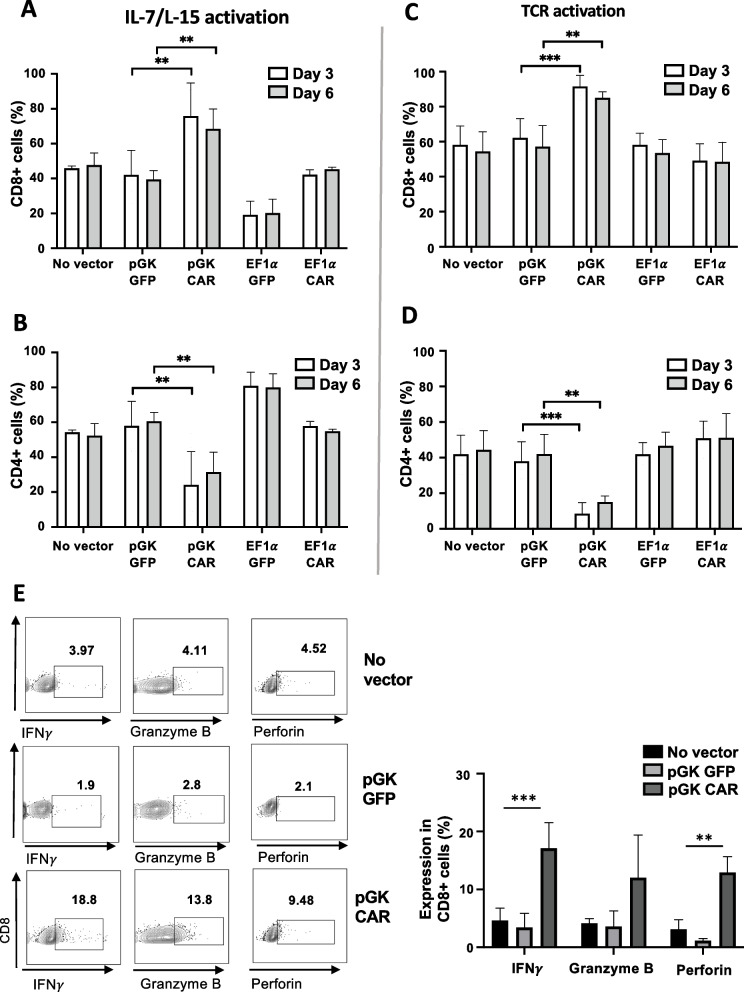
CD8-LVs mediated CD4-CAR expression by the PGK_prom_ outperformed EF1a_prom_ in CAR T cell activity. T cells were stimulated by IL-7/IL-15 or through the TCR and transduced as outlined in Fig. 2C with the indicated vectors at equivalent viral particle levels (10 ng p24). FACS analysis was performed on day 3 and 6 post-transduction to detect the % of CD8^+^ T cells (**A**,**C**) and CD4.^+^ T cells (**B**,**D**). (mean (SD); (**A**,**B**) No vector d3 *n* = 4, d6 *n* = 4; PGK-GFP d3 *n* = 5, d6 *n* = 3; PGK-CAR d3 *n* = 7, d6 *n* = 4; EF1a-GFP d3 *n* = 3, d6 *n* = 3; EF1a-CAR d3 *n* = 3, d6 *n* = 3 *n* = 4; **p < 0.01; (**C**,**D**), No vector d3 *n* = 7, d6 *n* = 6; PGK-GFP d3 *n* = 5, d6 *n* = 5; PGK-CAR d3 *n* = 7, d6 *n* = 4; EF1a-GFP d3 *n* = 3, d6 *n* = 3; EF1a-CAR d3 *n* = 3, d6 *n* = 3; **p < 0.01, ***p < 0.001; multiple t-test). (E) Six days post-transduction T cells were surface stained for CD8 followed by intracellular staining for IFNγ, perforin and granzyme B and analyzed by FACS. Representative FACS plots are shown and data are summarized in the histogram (mean ± SD, multiple t-test; no vector IFNγ *n* = 3, granzyme B *n* = 3, Perforin *n* = 3; pGK-GFP: IFNγ *n* = 4; granzyme B *n* = 4; perforin *n* = 3; pGK-CAR: IFNγ *n* = 5; granzyme B *n* = 4; perforin *n* = 3; **p < 0.01, ***p < 0.001)

In line with the high anti-CD4 CAR expression in the mCD8 T cell achieved from the PGK_prom,_ a clear and significant expansion of these mCD8 T cells and reduction of mCD4 T cells was detected (Supplementary Fig. 1). For both stimulation protocols (TCR and IL-7/IL-15) the PGK_prom_ driven CAR outperformed the EF1α_prom_ driven CAR mCD8-LVs in terms of CD8 expansion and CD4 T cell elimination since for the latter no significant difference with the corresponding GFP encoding vector or non-transduced T cells was detected (Fig. 3A-D). At equivalent vector doses, TCR activation resulted in higher CAR T cell activity than IL-7/IL-15 activation for the PGK_prom_ driven CAR mCD8-LVs (Fig. 3A, B versus C, D). Additionally, the high CAR expression by the PGK_prom_ in the mCD8 T cells induced upon encounter with the CD4 T cells a strong effector function confirmed by INFγ, granzyme B and perforin production by the cytotoxic CD8 + T cells (Fig. 3E). An important point is that CD4 is also expressed by other cell types besides CD4 + T lymphocytes, such as dendritic cells and macrophages. We therefore performed a co-culture experiment of GFP CD8-LV and anti-CD4 CAR CD8-LV transduced T cells and bone marrow derived macrophages (BMDM) in a 1/1 ratio (Supplementary Fig. 2A and B). After 3 days of co-culture no significant difference in the % of living macrophages was detected between macrophages cultured without T cells, in the presence of T cells expressing GFP or anti-CD4 CAR suggesting no major off-target effect of the antiCD4-CAR CD8 T cells on macrophages.

Concluding, the PGK_prom_ driven anti-CD4CAR mCD8-LVs outperformed the EF1α_prom_ driven CD4CAR mCD8-LVs in CD8 splenocyte expansion and CD4 splenocyte elimination.

### *Anti-CD4CAR* + *CD8 TILs eliminated malignant CD4 T cells in AITL lymphoma biopsies*

**Fig. 4:**
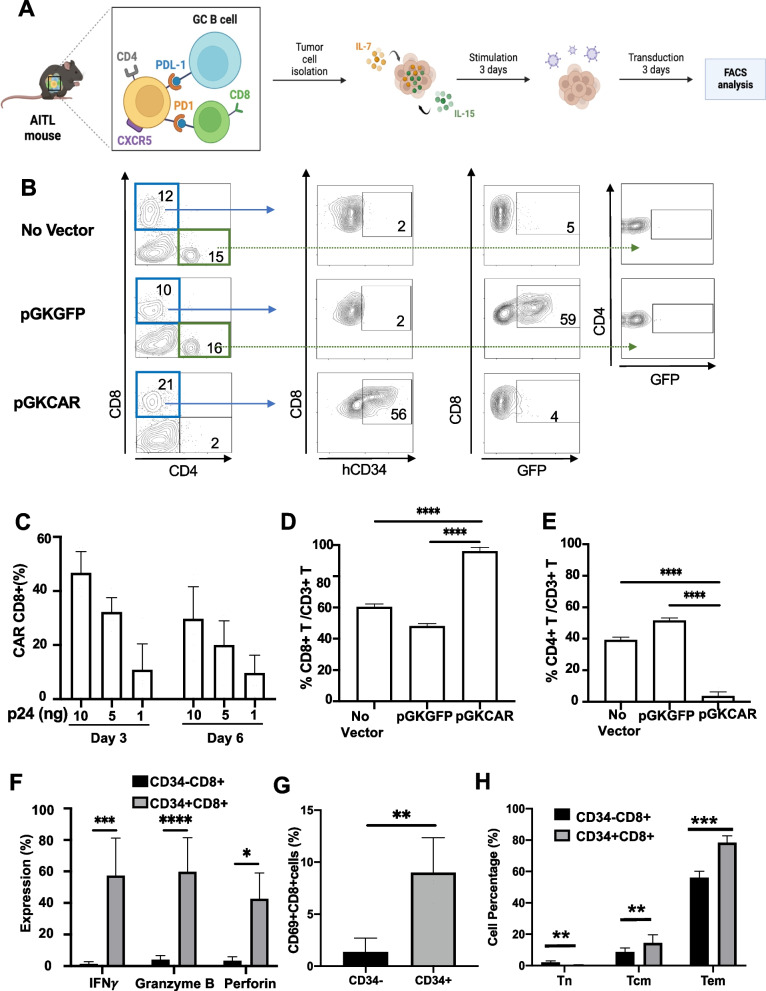
Anti-CD8-LVs encoding the CD4-CAR induced CD8 TIL expansion in murine AITL lymphoma biopsies and CD4 neoplastic T cell death. **A** Enlarged spleens from mice that developed AITL lymphoma (mAITL), were isolated and total tumor cells were put in culture in the presence of IL-7 and IL-15 as indicated in the workflow. Created by Biorender.com (**B**) FACS analysis was performed on day 3 to detect the % of hCD34 (mCD4CAR +) or GFP + expressing CD8 and CD4 T cells upon transduction with anti-CD4CAR- or GFP-encoding mCD8-LV respectively. Data are summarized as a histogram in (**C**) as mean (SD, *n* = 7). FACS analysis to determine the % of CD8 T cells (**D**) and CD4 T cells (**E**) 6 days post-transduction in the mAITL biopsies (mean (SD), No vector *n* = 3; PGK-CAR *n* = 8, PGK-GFP *n* = 3; one-way-Anova; ****p < 0.0001). **F** Six days post-transduction T cells were surface stained for CD8 and hCD34 followed by intracellular staining for INFγ, perforin and granzyme B and analyzed by FACS. Expression of these cytotoxic molecules are shown for CAR positive (hCD34 +) or negative (hCD34-) CD8 TILs. Data are summarized in the histogram (mean (SD), INFγ *n* = 6, Granzyme *n* = 6, Perforin *n* = 3; multiple student t-test; *p < 0.05, ***p < 0.001, ****p < 0.0001). **G** Three days post-transduction T cells were surface stained for CD8 and hCD34 followed by staining for CD69. The percentage of cells expressing CD69 activation marker is shown for CD8 TILs positive for the anti-CD4CAR (hCD34 +) or not (hCD34-) (mean (SD) *n* = 4, t-test,**p < 0.01). **H** Six days post-transduction T cells were surface stained for CD8 and hCD34 followed by staining for CD44 and CD62L. The percentage of naïve T cells (TN), central memory T cells (TCM) and effector memory is shown (TEM) (Mean (SD); *n* = 6, multiple t-test; ***p < 0.001,**p < 0.01)

The mCD8-LVs incorporating the anti-CD4CAR under the control of the PGK_prom_ was chosen for evaluation in murine AITL lymphoma, as it was the best performing candidate in WT splenocytes. We homogenized splenic lymphoma cells from aged plck-GAPDH mice developing AITL. These cell suspensions contained malignant CD4 + Thf-like cells, which express PD1 and CXCR5, and the associated tumor microenvironment consisting of germinal center (GC) B cells and CD8 TILs. We relied on IL-15/IL-7 stimulation of these cells to allow efficient transduction. The workflow of cell isolation, stimulation and transduction is shown in Fig. 4A. Three days upon transduction with the anti-CD4CAR CD8-LVs, the lymphoma cells were analysed by FACS for the presence of CD4 AITL cells and CD8 TILs. Up to 56% of the CD8 T cells expressed hCD34  (indicative for CAR expression) for the highest vector doses, while the GFP-encoding CD8-LV reached 59% (Fig. 4B and C). The anti-CD4CAR expression led to expansion of the CD8 TILs in the mAITL lymphoma (Fig. 4D), while the CD4 neoplastic Tfh-like cells were almost completely eliminated as compared to incubation with GFP encoding CD8-LVs or no vector (Fig. 4B and E). To confirm that the anti-CD4 CAR expressing CD8 TILs were the functional cytotoxic T cells, gating on the CD34 + and CD34- CD8 T cells in the tumors was performed (Supplementary Fig. 2), which revealed a significant increase in the cytotoxic molecules IFNγ, granzyme B and perforin exclusively in the CD34 + CD8 T cells indicating that the CAR positive CD8 + TILs upon encounter of their CD4 antigen reverted to cytotoxic T cells (Fig. 4F). In accordance the CD34 + CD8 T cells showed significant upregulation of the CD69 activation marker as compared to the CD34- CD8 T cells (Fig. 4G). Finally, we also confirmed a significant shift toward central memory (CMT, CD62L + CD44 +) and effector memory T cells (EMT, CD62L-CD44 +), while the naïve T cells were reduced (TN, CD62L + CD44 low) for the CD34 + CD8 T cell subset as compared to the CD34- CD8 T cells (Supplementary Fig. 4 and Fig. 4H).

In conclusion, anti-CD4 CAR CD8-LVs were able to convert CD8 T cells into powerful cytotoxic T cells that lysed the CD4 cancer T cells in murine AITL biopsies.

### In vivo* generation of anti-CD4CAR* + *CD8 T cells increased survival of the preclinical mAITL mice*

**Fig. 5:**
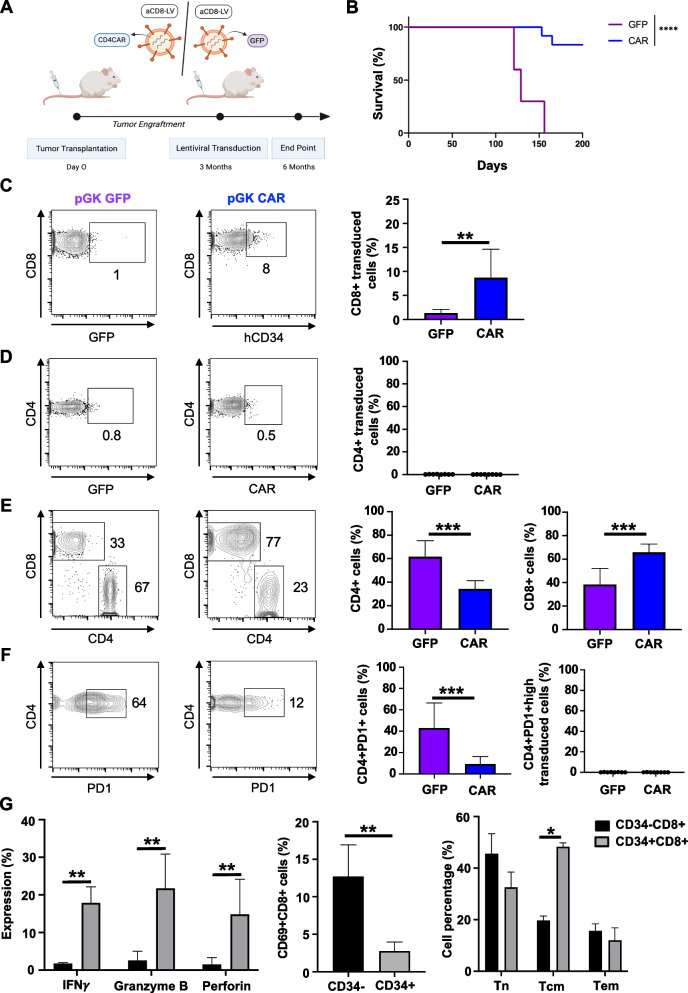
Anti-CD8-LVs encoding the antiCD4-CAR increased survival of the mAITL mice through elimination of neoplastic CD4 + PD1^high^ T cells (A) Splenic lymphoma cells from mAITL mice were injected intravenously into recipient NSG mice (*n* = 24). Three months upon engraftment, mice were treated with CD8-LV encoding GFP (*n* = 10) or anti-CD4CAR CD8-LVs (*n* = 10) by IV injection. Created with Biorender.com. Survival curves for both mouse groups are shown in (**B**). Mice were sacrificed at humane endpoint or 200 days post-transplant (****p < 0.0001, Mantel-Cox test). **C**,**D** FACS analysis of the percentage GFP + CD8 + and GFP + CD4 + cells in the PGK-GFP group and hCD34 + CD8 + and hCD34 + CD4 + cells in the PGK-CAR group at sacrifice. Representative FACS plots are shown and data are summarized in the histogram (mean (SD); *n* = 8 for PGK-GFP CD8-LV, *n* = 8 for PGK-CAR CD8-LV;t-test **p < 0.01) (**E**) FACS analysis of the percentage CD4 + and CD8 + cells in the indicated treatment groups at sacrifice; a representative FACS plot is shown and data are summarized in the histogram (mean (SD); *n* = 8 for PGK-GFP CD8-LV, *n* = 8 for PGK-CAR CD8-LV; t-test, ***p < 0.001). **F** FACS analysis of percentage of CD4 + PD1^high^ cells per total CD4 + T cells in the spleen and the percentage of GFP + and CD34 + CD4 + PD1.^high^ cells of the indicated treatment groups at sacrifice; a representative FACS plot is shown and data are summarized in the histogram (mean (SD); *n* = 8 for PGK-GFP CD8-LV, *n* = 8 for PGK-CAR CD8-LV; t-test, ***p < 0.001). **G** At sacrifice splenic T cells were surface stained for CD8 and hCD34 followed by intracellular staining for INFγ, perforin and granzyme B and analyzed by FACS. Expression of these cytotoxic molecules are shown for CAR positive (hCD34 +) or negative (hCD34-) CD8 TILs. Data are summarized in the histogram (right panel, mean (SD), INFγ *n* = 5, Granzyme *n* = 5, Perforin *n* = 5; multiple student t-test; **p < 0.01). Further, T cells were surface stained for CD8 and hCD34 followed by staining for CD69. The percentage of cells expressing CD69 activation marker is shown for CD8 TILs positive for the anti-CD4CAR (hCD34 +) or not (hCD34-) (middle panel mean (SD) *n* = 5, t-test,**p < 0.01). T cells were surface stained for CD8 and hCD34, CD44 and CD62L. The percentage of naïve T cells (TN), central memory T cells (TCM) and effector memory is shown (TEM) (Mean (SD); *n* = 5, multiple t-test;*p < 0.05)

Having confirmed neoplastic CD4 + T cell elimination in murine WT splenocytes and AITL biopsies encouraged us to evaluate the mCD8-targeted LVs encoding the anti-CD4CAR expression cassette in vivo in WT C57BL/6 mice. We performed an IV injection of the CAR-LV or GFP-LV in WT C57BL/6 mice with a follow-up of early and later timepoints in the blood for the CD8 + CAR + T cells and CD4 T cells as outlined in Supplementary Fig. 5A. A slow but continuous increase in CD8 + T cells and reduction in CD4 + T cells was detected, while there was a continuously increasing level of CAR + T cells over time (Supplementary Fig. 5B-C). At 17 weeks post vector injection we detected a significant increase in CD8 + and decrease in CD4 + C57BL/6 splenocytes and a significantly higher percentage of CAR-LV transduced CD8 T cells as compared to GFP-LV transduced cells. These result encouraged us to perform the in vivo CAR T cell generation in our preclinical mAITL model. For this purpose, we engrafted the mAITL lymphoma cells into a cohort of NOD/SCIDγc-/- (NSG) recipient mice as previously described [15, 17]. Since no sign of malignancy was detectable in the blood for AITL disease, we sacrificed one mouse at 6, 8 and 12 weeks of tumor cell injection. At 12 weeks upon engraftment a clear splenomegaly was confirmed and the presence of over 40% of PD1^high^ CD4 + malignant cells was detected in one mouse of the cohort (Supplementary Fig. 6). Therefore, we decided to inject subcutaneously IL-7 and IL15 followed by a single intravenous injection of mCD8-LVs encoding GFP or the anti-CD4CAR, both under the control of the pGK promoter (Fig. 5A). The CD8-LV coding for anti-CD4CAR resulted in a highly significant increased survival (80%) of the CAR group compared to the control group injected with CD8-LV coding for GFP (Fig. 5B). Indeed, the majority of the control treated mice reached endpoint much earlier. At sacrifice, the control group showed low level of CD8 T cell transduction (up to 2% GFP + cells). The level of genetically modified T cells was much higher in the CAR-LV injected group reaching up to 15% CD4CAR + CD8 T cells, while the CD4 T cells remained untransduced (Fig. 5C and D). In agreement, spleens from the CAR group contained twofold higher percentages of CD8^+^ T cells than the control group, while the CD4 + T cells followed an inverse pattern meaning significant CD4 + T cell elimination for the CAR treated group (Fig. 5E). Additionally, the CD4 + T cell subset was depleted for Tfh PD-1^high^ neoplastic cells in the CAR treated group (70%) as compared to the control group (Fig. 5F). Of utmost importance, for the CD4 + PD-1^high^ malignant cells and B cells transduction was undetectable (Fig. 5F and Supplementary Fig. 7). Additionally, we characterized the CD8 T cells in vivo by staining for activation (CD69) and verification of cytotoxicity and naïve/memory status of the CAR + and CAR- CD8 T cells at experimental endpoint (Fig. 5G). In agreement with the in vitro results (Fig. 4), the CAR + CD8 T cells though they are low in percentage, showed stronger activation and stronger cytotoxicity than their CAR- counterparts. Interestingly, more central memory but at endpoint no increase in effector memory cells was detected in the CAR-treated group.

In summary, CD8-LVs permitted the in vivo generation of functional anti-CD4CAR CD8 T cells, which reduced significantly the CD4 + lymphocytes from the tumors correlated with increased survival of the mAITL mice.

## Discussion

PTCL with a pronounced Tfh CD4 + component are cancers that are mostly detected only at late stage of disease. Currently, new proposed treatments do not increase the life expectancy of these patients compared to classical chemotherapy. We engineered a lentiviral vector incorporating a CAR against the CD4 receptor present on the PTCL malignant CD4 + cells. To increase safety, we avoided expression of the CAR on the CD4 malignant cells by exposing at the surface of the anti-CD4 CAR LVs a CD8-targeting ligand. Simultaneously, CD8-specific CAR delivery prevented CAR T cell fratricide as it was described for CD4 CAR T cells. Our approach assured specific transduction of CD8 T cells resulting in high level anti-CD4 CAR expression by the CD8 T cells present in lymphoma biopsies of a preclinical mAITL mouse model. These CAR expressing TILs gained a cytotoxic phenotype and efficiently eliminated the CD4 Tfh cells in the mAITL biopsies as well as in vivo in a AITL preclinical mouse model.

Although AITL and CD4 + Tfh-like PTCL are the most frequent PTCL entities, the number of patients is rare and far from sufficient to conduct conclusive clinical trials. It is therefore very challenging to evaluate new therapies for AITL, for which patient biopsies are very scarce and only a few AITL or Tfh-like PTCL patients can be enrolled in clinical trials. As expected, these trials including only a few PTCL patients might not give a clear-cut outcome and conclusion in terms of therapeutic benefit for these cancer patients. Fortunately, preclinical mouse models for AITL are now finally available [11–14, 44]. Today, several genetic mouse models mimic Tfh PTCL/AITL in terms of clinical, pathological, histological, transcriptional, genetic and immunophenotypic features: they are based on the knockout of Tet2 and overexpression of RhoAG17V [11, 44], based on Tet2 and IDH2 mutations [13] and our transgenic mouse model is based on GAPDH overexpression in the T cell lineage [15]. Up to now, these models allowed to unravel signaling pathways such as mTOR or NF-κB pathways [11, 15] and the dependance of AITL neoplastic cells on choline metabolism [17]. Inhibiting these pathways pointed towards new therapies. Here we used our mAITL preclinical model to validate the generation of anti-CD4 CAR T cells in vivo exclusively expressed by the CD8 T cells in the AITL tumor environment. Aberrant transduction of the malignant CD4 T cells might lead to high toxicity and risk of developing a blood cancer as already shown in one case of ex vivo CAR T cell generation [22]. We did not detect unspecific transduction and CAR expression in the neoplastic CD4 T cells. This means that in terms of safety, these CD8-targeted LVs might also be of benefit in the case of ex vivo CAR T therapy since contamination of the CD8-purified cell subset with CD4 + T cells cannot be entirely excluded when using polytropic VSV-G-LVs.

Importantly, targeting a specific cancer cell type without inducing a severe side-effect on healthy cells requires an in-depth knowledge of its surface markers, which can serve as targets for immunotherapies. If some healthy cells are modified this is called on-target off-target effects because certain cancer epitopes are partially expressed on healthy cells. In the case of AITL choosing the right receptor is not straight forward. The CD4 + Tfh cell, the malignant driver of AITL highly expresses surface markers such as CD4, CXCR5, PD-1, ICOS and CD40L that determine its Tfh-like phenotype [16]. Indeed, the CD4 + Tfh cell survival relies on ICOS surface expression, which is induced by TCR-stimulation [45] and ICOS plays a crucial role in Tfh cell localization in the germinal center (GC) [46]*,*where through T-B cell interaction B cells are turned into potent antibody producing cells. Of note, AITL Tfh CD4 cells show very high similarity to healthy Tfh cells, also in their T-B-cell interaction through ICOS-ICOSL binding [47]. Thus, ICOS was considered a possible target for antibody based immunotherapy but still could eliminate healthy Tfh and B cells. As ICOS, PD-1 is highly expressed by Tfh cells in AITL. While PD-1 is known as inhibitory in immune cells, PD-1 plays an important role in Tfh development and activity [48]. Within the GC, PD-1 expression controls Tfh and B-cell proliferation and survival [49]. In addition, PD-1 was found to inhibit the cytotoxic function by interacting with PDLs expressed on anti-tumoral cells such as natural killers and cytotoxic CD8 cells in lymphoma [50]. Taken together, PD-1 presented an important candidate target for antibody-based immunotherapy in AITL. In this context, we previously used an anti-PD-1 antibody in combination with a non-canonical NF-kB inhibitor to treat mice bearing AITL tumors. Survival increased up to 70% compared to non-treated mice [15]. These are encouraging results, but the same study showed only 40% survival upon anti-PD-1 immunotherapy as a single treatment. It was also believed that in PTCL PD-1 itself could be a tumor suppressor [51]. Indeed, in some mouse studies, anti-PD-1 treatment caused violent progression of adult T cell lymphomas [52] and clinical trials including PTCL showed only very low activity upon anti-PD-1 single treatment [53, 54]. However, both ICOS and PD-1 cannot be chosen for CAR T cell generation in vivo since they are expressed on both CD4 and CD8 T cells which will lead to fratricide, which is the phenomenon we avoid here by targeting CD8 T cells, exclusively. Further, CD30 surface expression on AITL cells can be detected in up to 43% of the patients [42] and has a pleiotropic effect on cell growth and survival [55]. To explore the CD30 potency as a target, clinical trials are exploring anti-CD30CAR T cells for AITL patients and other T cell lymphomas (NCT04008394). Finally, CD52 is considered as immunotherapy target but it is widely expressed by the immune system so not exclusive on the malignant cells [56]. CD52 is used as target for T and NK cell malignancies including AITL, in which CD52 is highly expressed [57] but no results are communicated up to now from clinical trials.

In vitro the generated anti-CD4 CAR CD8 T cells were able to eliminate the CD4 T cells in WT C57BL/6 splenocytes as well as in mAITL lymphoma biopsies. In vitro (Fig. 4) as well as in vivo (Fig. 5), the CAR + CD8 T cells, though they are low in percentage, showed stronger activation and stronger cytotoxicity than their CAR negative counterparts. However, more central memory but not effector memory cells were detected in CAR + versus CAR- CD8 T cells at the endpoint (Fig. 5). Interestingly, we also detected a reduction of the exhaustion marker PD-1 on the total CD8 T cells in the CAR-LV treated group compared to GFP-LV group indicating that the CD8 T cells in the tumor regained their effector potential.

The elimination of CD4 + T cells was more robust upon TCR activation than IL-7 /IL-15 stimulation. This could be explained by fact that the CD4 receptor is upregulated upon TCR stimulation [58] and therefore anti-CD4 CAR binding to the CD4 T cells is more efficient, inducing their elimination more effiently. Likewise, this low-level expression of CD4 on resting and mildly stimulated T cells might explain the slow and gradual decrease of the CD4 + T cells in vivo in WT mice in the blood circulation but also at sacrifice in the mAITL treated mice. Importantly, at sacrifice we clearly demonstrated that the malignant CD4 PD1^high^ cells were preferentially eliminated as compared to healthy CD4 T cells in the mAITL mice. Unfortunately, a follow-up of the CD8 T cell expansion, the percentage of CAR + CD8 + T cells and the malignant CD4 T cell depletion by blood punction at several time-points is not possible in the mAITL mouse model. The lymphoma develops in the spleen and lymph nodes and no traces or very few malignant CD4 T cells and CD8 T cells can be detected in the blood circulation. This phenotype closely mimics AITL patients, for whom no to very few malignant CD4 + PD1^high^ T cells can be found in the circulation, which is the reason why the malignancy is only detected in late stages. Moreover, though we detected that TCR stimulation before transduction gave superior results in terms of transduction of CD8 T cells and CD4 T cell elimination in WT splenocytes, we cannot use TCR stimulation in a clinical setting, while IL-15 and IL-7 could be envisaged. In addition, we have shown that that TCR stimulation in our in vitro experiment induced already severe cell death in the CD4 + PD1^high^ malignant T cells. This can be attributed to the fact that CD4 + PD-1^high^ AITL cells mainly use mitochondrial respiration (Oxidative phosphorylation) as metabolic pathway to fuel their energy requirements [17]. Since TCR stimulation forces T cells towards a glycolytic metabolism, the malignant CD4 + AITL T cells, which are addicted to mitochondrial respiration shift to glycolysis, which induces their cell death.

Finally, AITL Tfh cells are derived from CD4 + T cells. Thus, using anti-CD4-CAR T cells in AITL patients as used in this study, might represent a promising strategy, even though healthy CD4 T cells will also be depleted and on the long term this approach can result in CD4 T cell elimination and immunosuppression. In the case of anti-CD19CAR T cells, healthy B cell depletion seem to be well tolerated in B cell malignancy, however, there are no sufficient data available to extrapolate this to T cell lymphomas. In contrast to pan T cell targeted CARs such as anti-CD7 CARs, the CD8 T cell population is not targeted in our CAR therapy and their persistence might decrease the risk of infections compared to other total T-cell directed CAR strategies. Nevertheless, the anti-CD4CAR CD8 T cells will target all CD4 T cells, malignant or healthy, which might lead to some immunosuppression in the patients. To overcome this problem, we can envisage several solutions to eliminate specifically the CAR T cells in vivo. One strategy could be to co-express with the CAR a receptor or a peptide. Administrating a monoclonal antibody (immunotherapy) specific for this receptor or peptide will eliminate the CAR T cells. In the case of AITL, CD20 might be a good candidate for co-expression on the CAR T cells because of a dual effect. Injection of an anti-CD20 monoclonal antibody might in this case eliminate the anti-CD4 CAR T cells as well as mature B cells, which are part of the tumor microenvironment and are important for the survival of the malignant CD4 T cells. Anti-CD20 treatments such as rituximab are already used in the clinic for both T and B cell lymphoma. A second strategy relies on co-expression of a ‘suicide gene’ together with the CAR such as thymidine kinase. A prodrug (ganciclovir) will be transformed into a toxic agent by thymidine kinase and will induce death of CAR T cells expressing this enzyme [59]. Pinz et al. [41, 42, 60] constructed antiCD4 CAR T cells anti-CD4-CAR-NK cells that eliminated specifically and robustly diverse ex vivo CD4 + human T-cell leukemia and lymphoma cell lines upon transfer of the these ex vivo expanded CAR T or NK cells in vivo. These preclinical results are encouraging for anti-CD4 CAR NK therapy use in case of all CD4 + T-cell malignancies and in particular for AITL [60]. The advantage of an anti-CD4 NK cell application might be that CAR NK-cells as compared to CAR T cells are short lived and do not lead to extended healthy CD4 T cell immunosuppression in the patients. However, this still needs to be consolidated.

Several findings strongly suggest that our in vivo CAR T cell generation therapy in the mAITL mouse model might be translated to patients. We have previously shown that we were able to generate via IV injection of CD8- or CD3- targeted LVs in humanized or xenograft mouse models, human CAR T cells in vivo that were able to eliminate healthy B cells or lymphoma B cells [27, 28, 30, 33]. However, one needs to be aware that these mouse models are not fully immune competent [61, 62] since for example humanized NOD/SCID γc-/- (NSG) mice do not develop functional human myeloid cells which might eliminate the vectors of CAR + T cells before they can expand and encounter the cancer antigen. In addition, several murine cytokines are not functional on human T cells. Another preclinical model, the patient derived xenograft (PDX) mouse model for AITL is not mimicking closely the T cell lymphoma since upon transplantation of human AITL biopsies into NSG mice the CD8 T cells and B cells do not engraft anymore and only the CD4 + PD1^high^neoplastic cells persist. For this reason, we used here an mAITL preclinical mouse model for the generation of CAR T cells since they contain in the lymphoma microenvironment next to the malignant CD4 + T cells, all the key immune players such as B cells, CD8 T and myeloid cells such as follicular DCs [15]. Translation to humans of our in vivo CAR T cell generation strategy is also encouraged by a recent clinical Phase I trial for treatment of T cell lymphoma patients demonstrating that ex vivo generated anti-CD4 CAR T cells resulted in complete remission in the first three patients treated without development of severe side effects [42].

Of importance for our in vivo strategy, one possibility to avoid immune responses against the LVs in vivo is by protecting these LVs through surface expression of CD47, which induces a ‘do not eat me’ signal to macrophages and avoids phagocytosis [63, 64].

## Conclusions

In vivo gene therapy with the CD8-retargeted LVs encoding for a CAR, might result in a huge benefit in terms of costs. We change the treatment from a highly intensive personalized therapy consisting of extracting the T cells from the patients, transducing them ex vivo and expanding them before reinfusion to a simple injection of a CAR encoding vector into the blood stream. Except for the vector production, all the other labor intensive and costly steps in the CAR T cell generation are not required anymore. Another advantage is that the CAR T cells generated in vivo in their physiological environment might not have such an altered phenotype as compared to their ex vivo expanded counterpart CAR T cells.

Concluding, here we engineered LVs that specifically target CD8 T cells in vivo to introduce an antiCD4 CAR expression cassette. This transformed the CD8 T cells into powerful cytotoxic CD8 T cells, which expanded in vivo upon encounter with the CD4 + T cells. In addition, the anti-CD4CAR expressing CD8-LVs were capable of eliminating the malignant CD4 T cells in the tumor bearing mAITL mouse model and increasing their survival.

## Supplementary Information

Supplementary Material 1.

## Data Availability

Material generated in this study are available upon request from the corresponding author (els.verhoeyen@unice.fr). The the CD8-MV targeted envelope is available from C.J. Buchholz. All data generated or analyzed during this study are included in this published article and its supplementary information. Raw data are available upon request.

## Abbreviations

Tfh: Follicular helper T
AITL: Angioimmunoblastic T cell lymphoma
PTCL: Peripheral T cell lymphoma
GAPDH: Glyceraldehyde-3-phosphate dehydrogenase
LV: Lentiviral vector
Gps: Envelope glycoproteins
MV: Measles virus
NiV: Nipah virus
ICOS: Inducible T-cell co-stimulator
PD-1: Programed death 1
TRBC1: T cell receptor β-chain constant region 1
mCD8-LV: Murin CD8 receptor targeted LVs (mCD8-LVs)
EF1α: Elongation factor 1 α
PGK: Phosphoglycerate kinase 1
NSG: NOD/SCIDγ-/- mice
TCR: T cell receptor
MFI: Mean fluorescence intensity
GC: Germinal center
