# Supplementary material for: In vivo CAR T cell therapy against angioimmunoblastic T cell lymphoma
Source: J Exp Clin Cancer Res. 2024 Sep 14;43:262. doi: 10.1186/s13046-024-03179-5 (PMC11401350; doi:10.1186/s13046-024-03179-5)
Supplement: Supplementary file 1 — Supplementary Material 1. [file 13046_2024_3179_MOESM1_ESM.pdf]

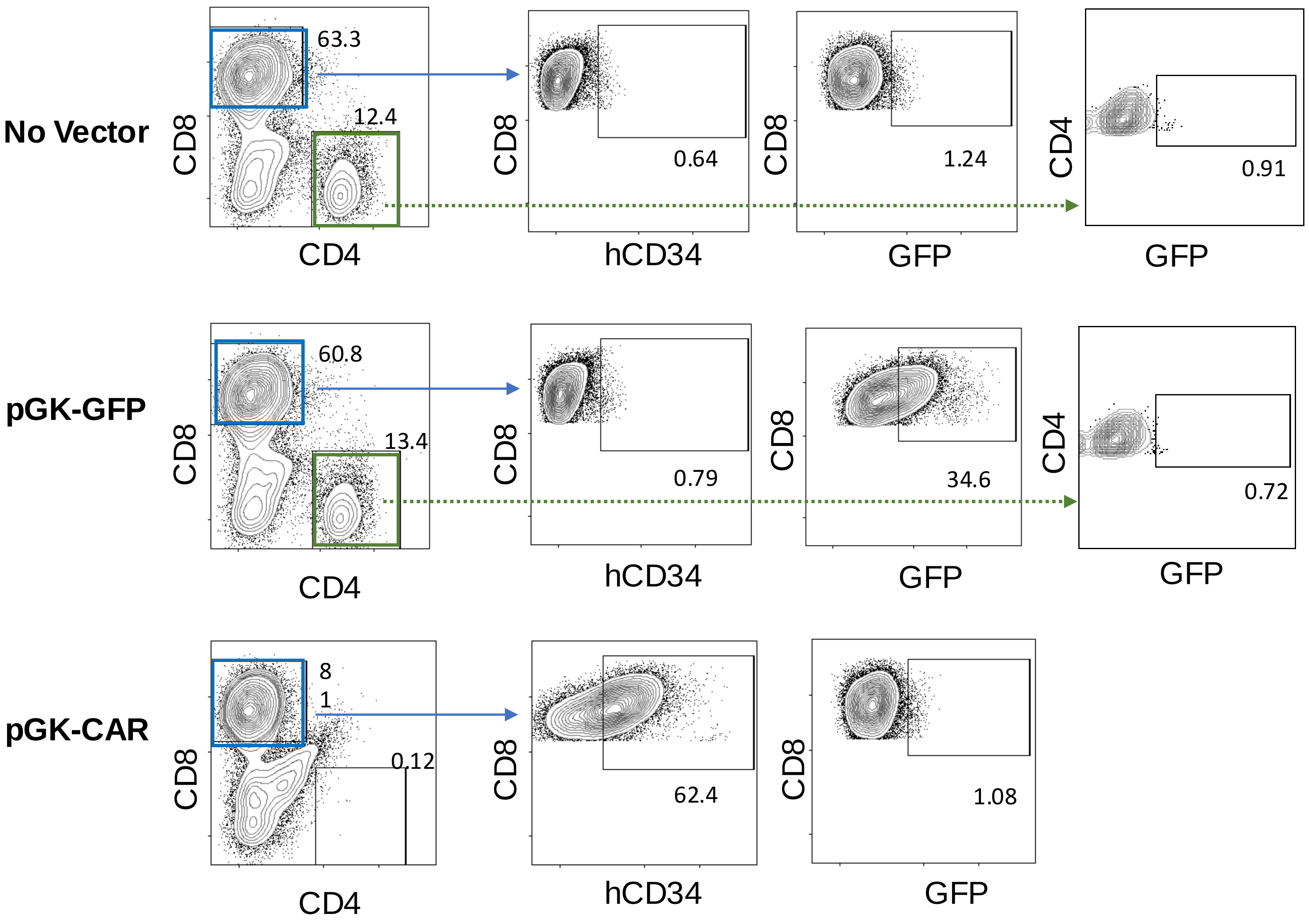

Supplementary Figure 1. mCD8-LVs allows high level mCD4CAR or GFP expression from the PGK promoter in CD8+ T cells

C57/bl6 splenocytes were stimulated by IL-7/IL-15 and transduced as outlined in Figure 2. T cell transduction with the indicated vectors at equivalent viral particle levels (10ng p24) was performed. FACS analysis was performed to detect the % of hCD34+ or GFP+ expressing CD8 and CD4 T cells 3 days post-transduction with anti-CD4CAR- or GFP-encoding mCD8-LV.

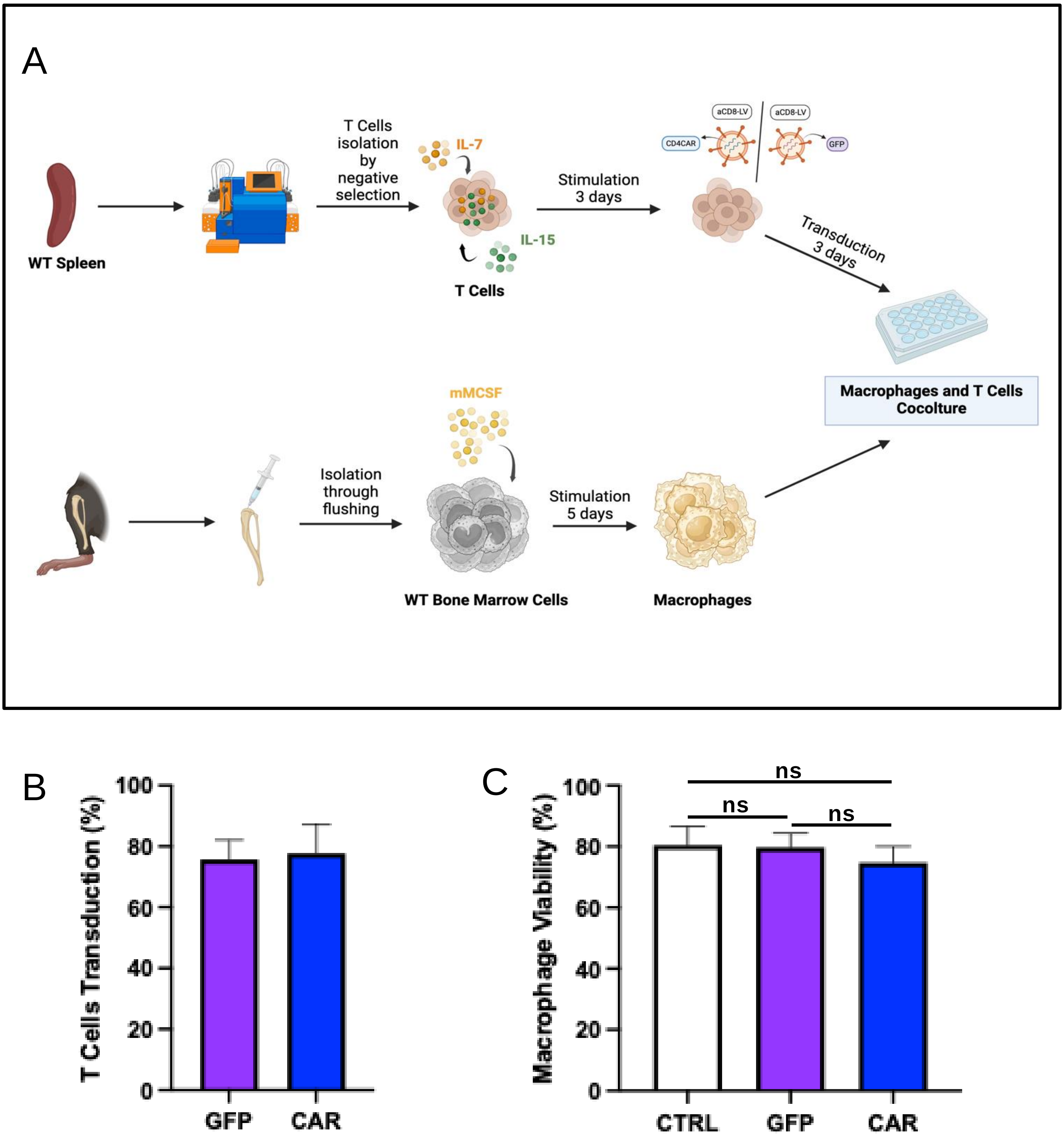

Supplementary Figure 2. Anti-CD4CAR CD8 T cells do not show off-target effect on macrophagegs in a T cell/macrophage co-culture

(A) Splenic T cells and BM cells were isolated from wt C57/bl6 mice. T cells were stimulated by IL-7/IL-15 and transduced for 3 days with anti-CD4CAR- or GFP-encoding mCD8-LV. T cell transduction with the indicated vectors at equivalent viral particle levels (10ng p24) was performed. In parallel, BM cells were differentiated in the presence of macrophage colony stimulating factor (M-CSF) into macrophages for 6 days and untransduced (CTRL) or transduced T cells were put in co-culture with macrophages at a ratio 1/1 (B) FACS analysis was performed to detect the % of hCD34+ or GFP+ expressing CD8 T cells 3 days post-transduction with anti-CD4CAR- or GFP-encoding mCD8-LV. (C) FACS analysis using DAPI staining to determine % cell survival of macrophages in the co-culture. All data are shown as mean (SD), untransduced CTRL (n=4) pGK-GFP (n=6) or pGK-CAR (n=9); student t-test; ns = not significant.

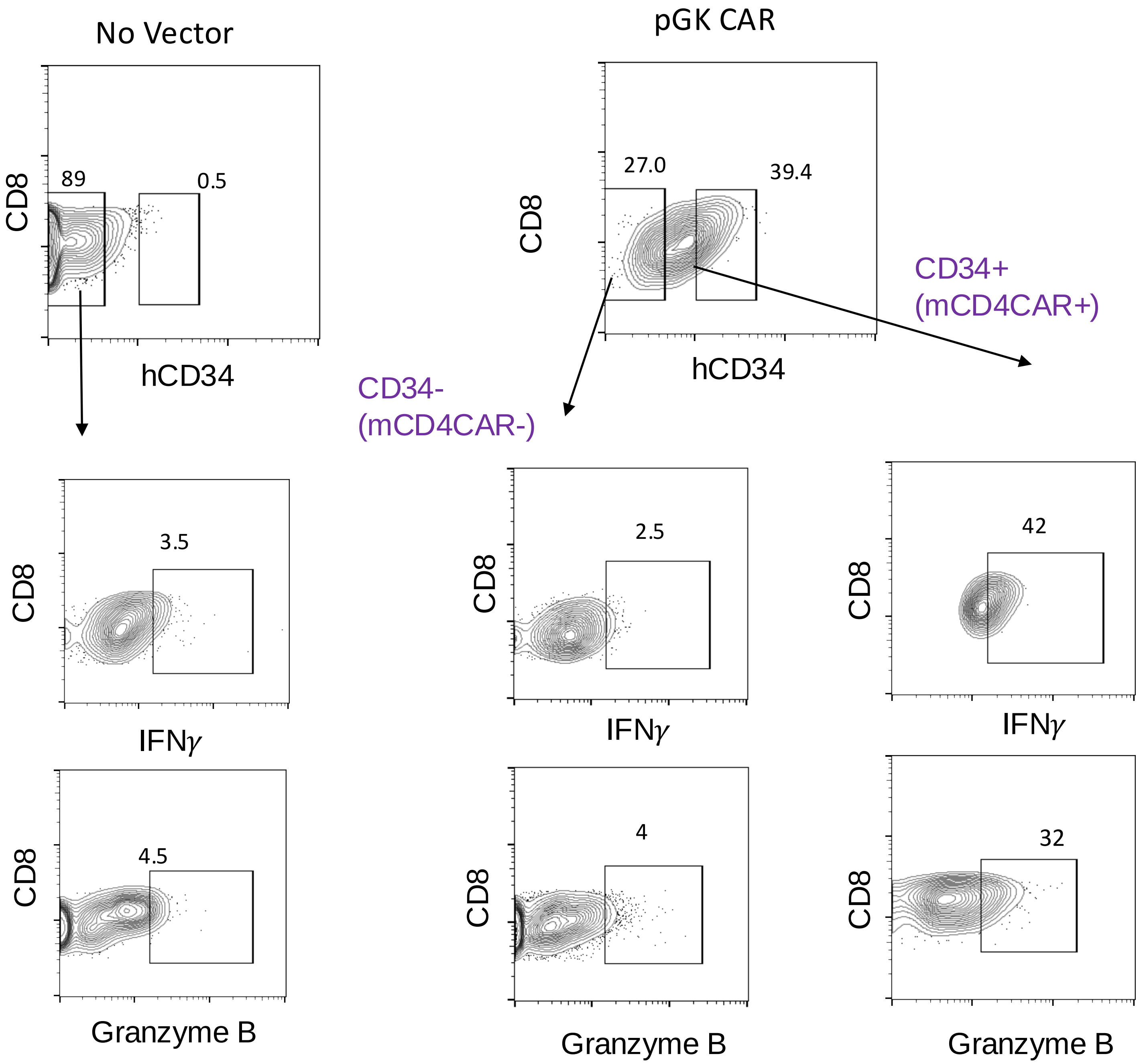

Supplementary Figure 3. CD8 TILs in the mAITL environment become cytotoxic when they express the mCD4CAR

Enlarged spleens from mice that developed AITL lymphoma (mAITL), were isolated and total tumor cells were put in culture in the presence of IL-7 and IL-15 and transduced as indicated in the workflow of Figure 4A. Six days post-transduction T cells were surface stained for CD8 and hCD34 followed by intracellular staining for INFγ and granzyme B and analyzed by FACS. Expression of these molecules is shown for CD8 TILs positive for the anti-CD4CAR (hCD34+) or not (CD34-) and non-transduced (No vector) splenocytes. Data are representative of n=6.

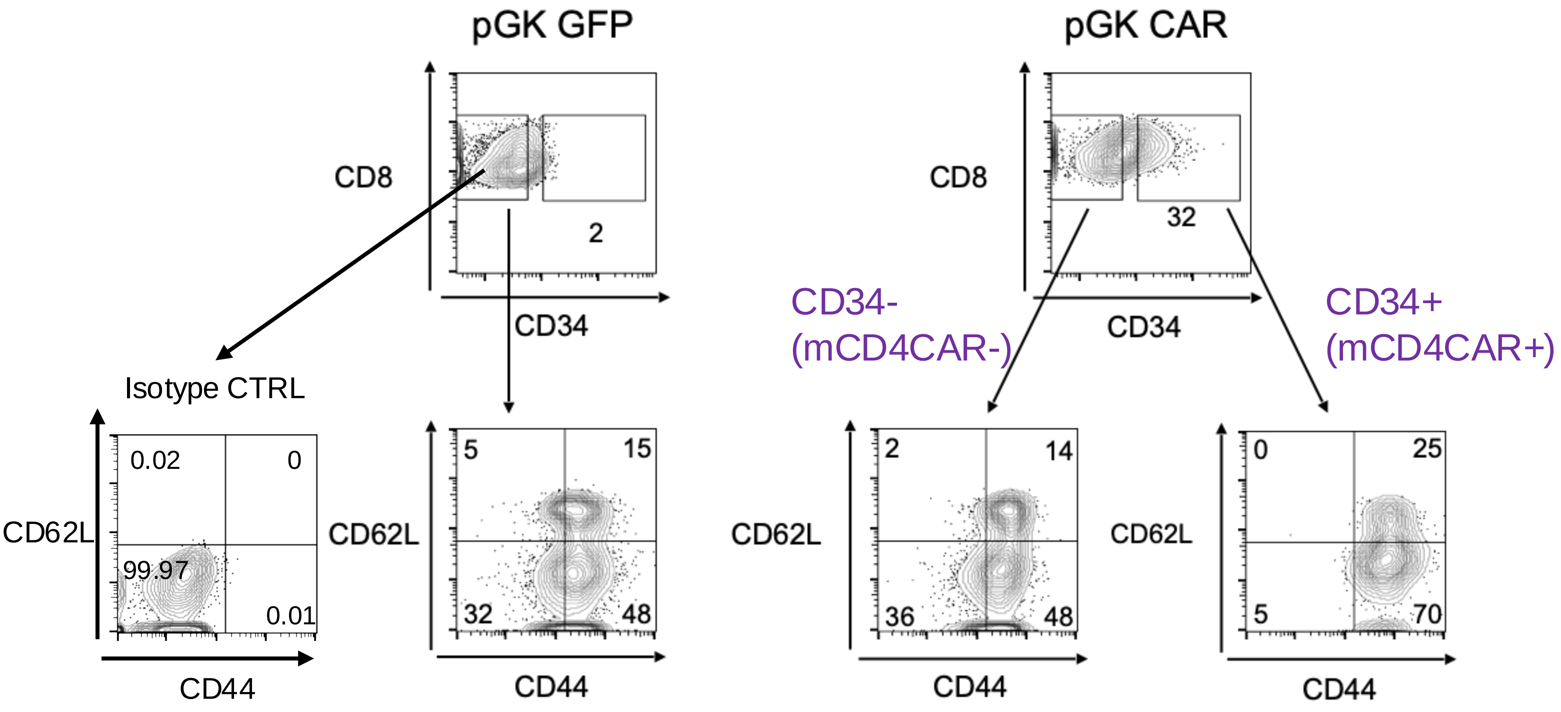

Supplementary Figure 4 Anti-CD8-LVs encoding the CD4-CAR induced CD8 TIL to shift to a central and effector memory phenotype

Enlarged spleens from mice that developed AITL lymphoma (mAITL), were isolated and total tumor cells were put in culture in the presence of IL-7 and IL-15 and transduced as indicated in the workflow of figure 4A. Six days post-transduction with anti-CD8-LVs encoding the anti-CD4-CAR, T cells were surface stained for CD8, hCD34, CD44 and CD62L. The percentage of naïve T cells (CD62L+CD44low), central memory T cells (CD62L+CD44+) and effector memory (CD62L-CD44+) is shown for the CD34+ and CD34- subsets. As a control the GFP encoding anti-CD8-LVs are shown.

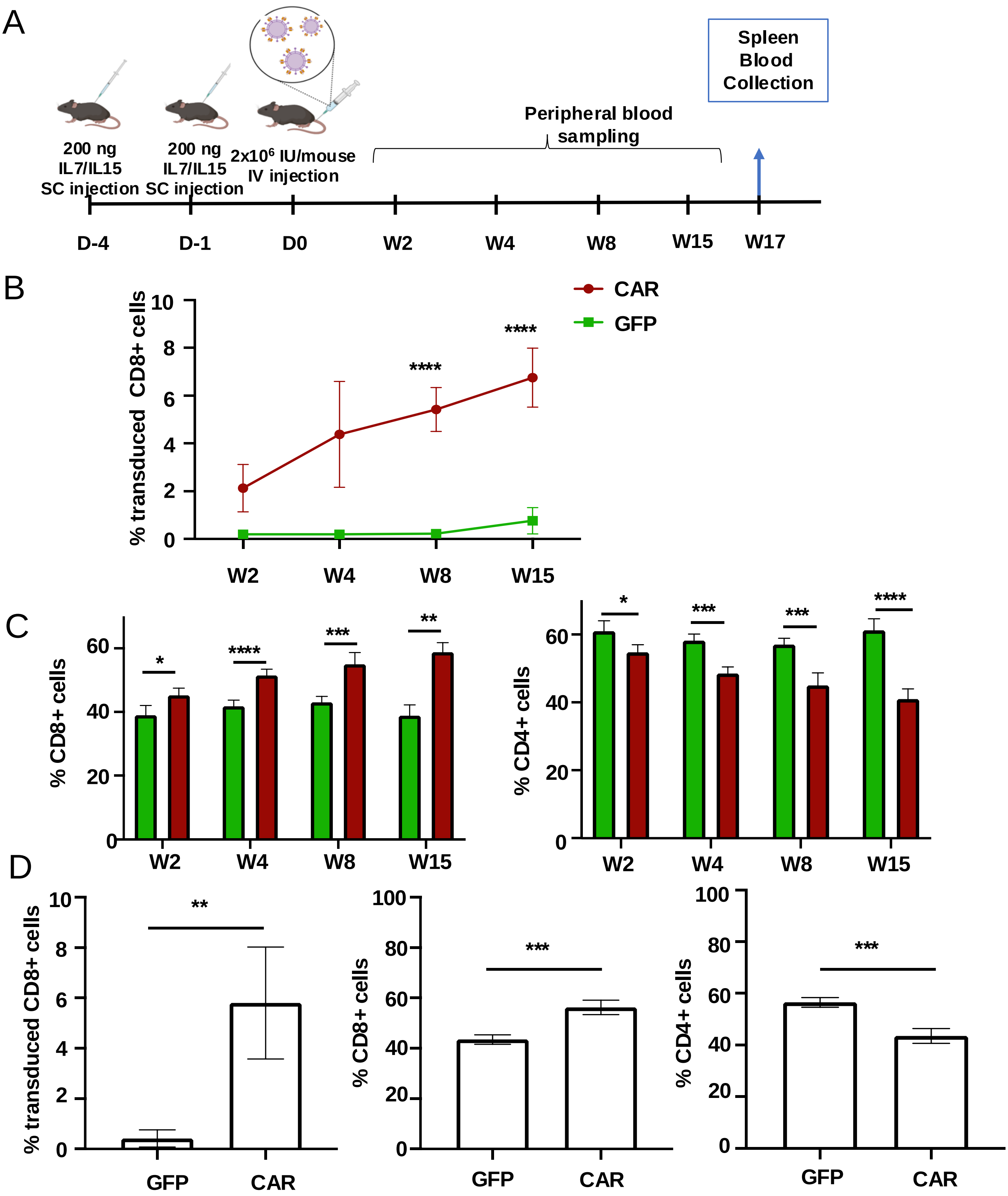

Supplementary Figure 5: In vivo injection of CD8-LV coding for anti-CD4-CAR gradually deplete CD4 T cells in wt C57BL6 mice.
(A) Layout of the in vivo experiment. C57BL6 mice were injected intravenously with IL-7 and IL-15 followed by IV injection with CD8-LVs coding for pGK-GFP (n=6) or pGK-CAR (n=6). D=day (B) FACS analysis of the % transduced CD8 T cells in the blood at week (W) 2, 4, 8 and 15 post-vector injection for the indicated treatment groups (pGK-GFP and pGK-CAR). (C) FACS analysis of the percentage of CD4+ and CD8+ T cells in the indicated treatment groups at week 2, 4, 8 and 15. (D) FACs analysis of the transduction and percentage of CD4+ and CD8+ splenocytes in the indicated treatment groups at sacrifice (week 17 post vector injection). All data are shown as mean (SD), for pGK-GFP (n=6) or pGK-CAR (n=6); student t-test; *p<0.05, **p<0.01***p<0.001, ****p<0.0001)

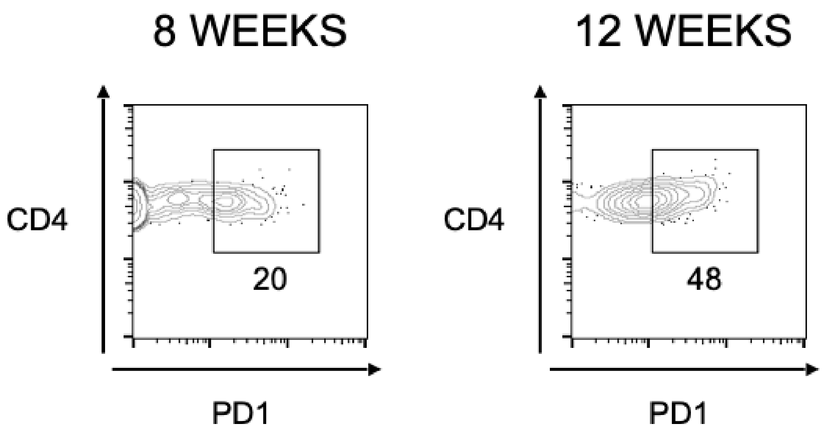

Supplementary Figure 6: Follow up of mAITL tumor engraftment in NSG recipient mice. Splenic lymphoma cells from mAITL mice were injected intravenously into recipient NSG mice (n=24). To confirm efficient lymphoma development, some mice were sacrificed at week 8 and 12 post lymphoma cell injection. FACs staining of the malignant splenocytes for CD4 and PD1 is shown for the 2 timepoints.

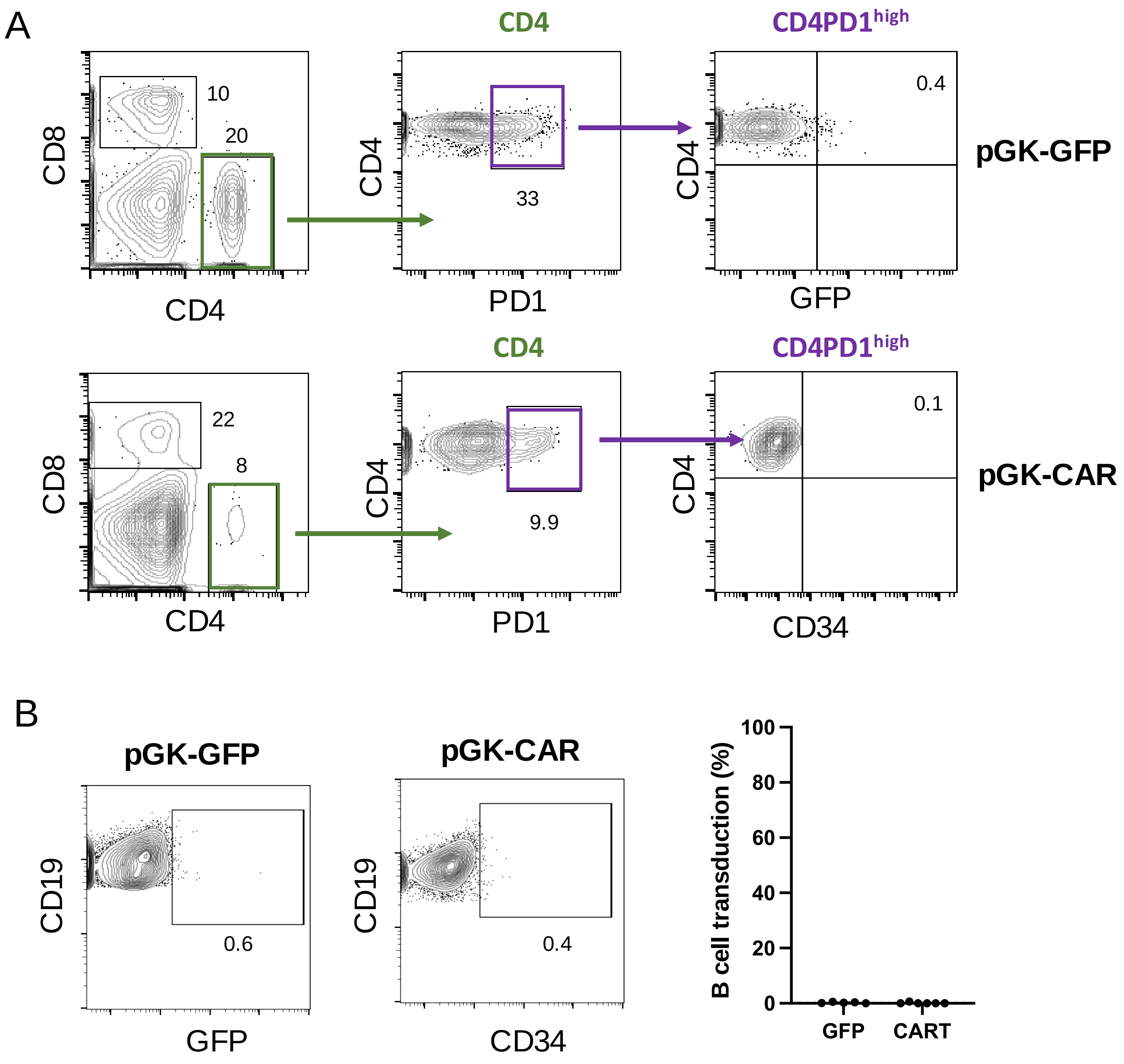

Supplementary Figure 7: CD8-LVs do not show off-target transduction in CD4+PD1^{high} lymphoma cells nor B cells in mAITL mice

(A) FACS analysis of the transduction of malignant CD4+PD1^{high} in the preclinical mAITL mice for the indicated treatment groups. FACS analysis is representative for n=5. Histogram summarizing the data in shown in Figure 5.

(B) FACS analysis of the transduction of B cells in the preclinical mAITL mice for the indicated treatment groups. FACS analysis is representative for n=5. Histogram summarizing the data.

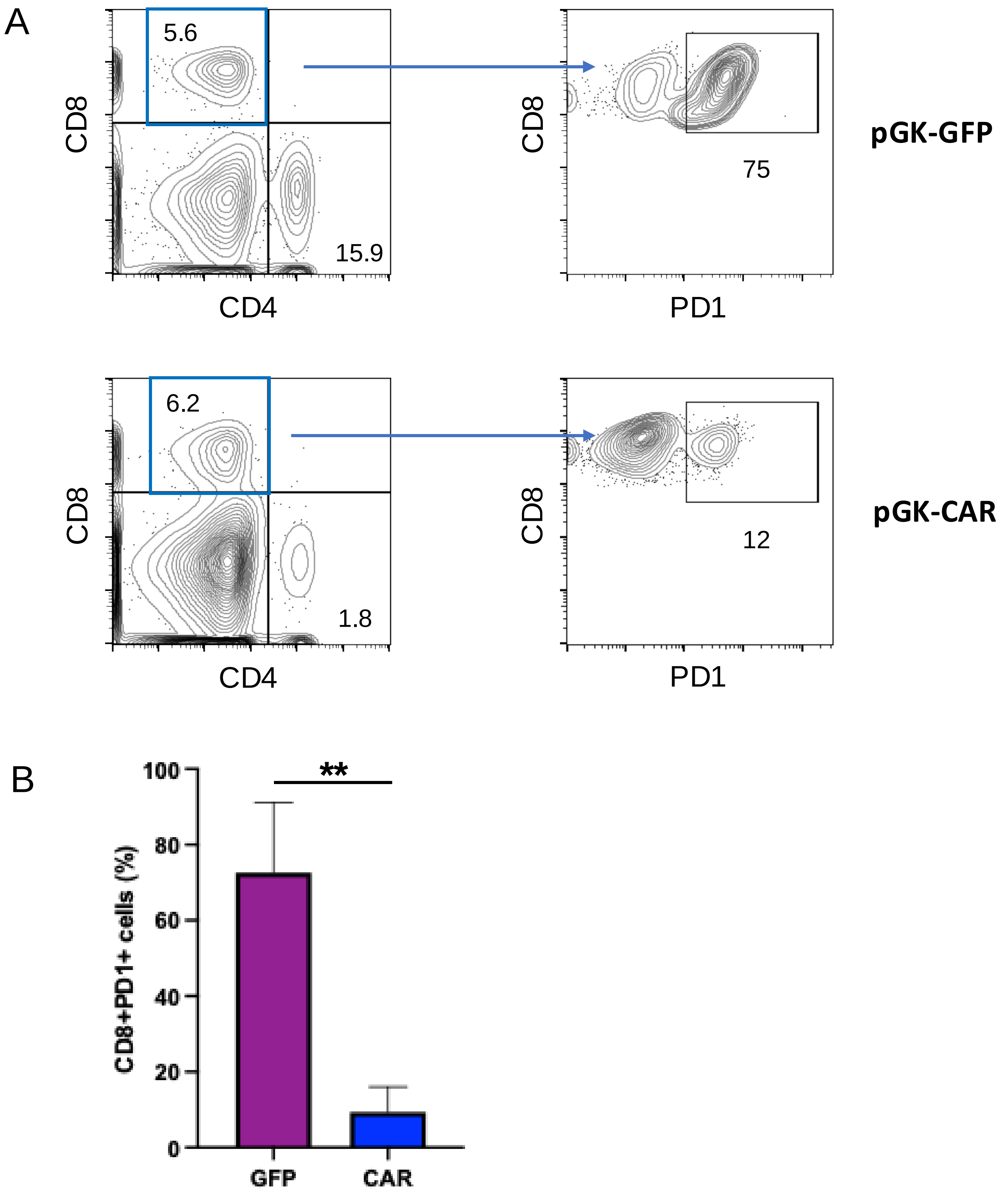

Supplementary Figure 8: mAITL mice injected with anti-CD4 CAR encoding CD8-LVs are characterized by reversion of the CD8 exhaused phenotype to effector cells.

(A) FACS analysis of PD1 exhaustion marker expression on CD8 T cells in the preclinical mAITL mice for the indicated treatment groups. FACS analysis is representative for n=5. (B) Histogram showing the PD1 exhaustion marker expression on CD8 T cells in the preclinical mAITL mice for the indicated treatment groups (mean (SD), for pGK-GFP (n=5) or pGK-CAR (n=5); student t-test; **p<0.01)
